# Mechanical Properties of Experimental Composites with Different Photoinitiator

**DOI:** 10.1055/s-0041-1731929

**Published:** 2021-08-24

**Authors:** Luis Felipe Marques de Resende, Anderson Catelan, Kusai Baroudi, Alan Rodrigo Muniz Palialol, Alexandre Marques de Resende, Ana Carolina Andreucci, Rayssa Ferreira Zanatta, Priscila Christiane Suzy Liporoni

**Affiliations:** 1 Department of Restorative Dentistry, University of Taubaté, Taubaté, São Paulo, Brazil; 2 Department of Dentistry, Faculty of Health Sciences, University of Western São Paulo, Presidente Prudente, São Paulo, Brazil; 3 School of Dentistry, University of Taubaté, Taubaté, São Paulo, Brazil; 4 Department of Restorative Dentistry, Piracicaba Dental School, University of Campinas, Piracicaba, São Paulo, Brazil; 5 Department of Restorative Dentistry, Federal University of Juiz Fora, Juiz de Fora, MG, Brazil

**Keywords:** resin composites, hardness, elastic modulus, photoinitiators

## Abstract

**Objective** The effect of different photoinitiators on mechanical properties of experimental composites was evaluated.

**Materials and Methods** Resin composites were formulated by using a blend of bisphenol A-glycidyl and triethylene glycol (50/50 wt%) dimethacrylate monomers, and 65 wt% of barium aluminium silicate and silica filler particles. Photoinitiators used were 0.2% camphorquinone (CQ) and 0.8% co-initiator (DMAEMA); 0.2% phenyl-propanedione and 0.8% DMAEMA; 0.1% CQ + 0.1% phenyl propanedione and 0.8% DMAEMA; 0.42% mono(acyl)phosphine oxide (MAPO); and 0.5% bis(acyl)phosphine oxide (BAPO). Specimens ( *n* = 10) were light cured by using a multiple-emission peak light-emitting diode for 20 seconds at 1,200 mW/cm ^2^ of irradiance and Knoop hardness and plasticization, depth of cure, flexural strength, and elastic modulus were evaluated. Data were statiscally analyzed at significance level of α = 5%.

**Results** Experimental composites containing MAPO and BAPO photoinitiators showed the highest values of flexural strength, elastic modulus, top surface hardness, and lower hardness reduction caused by alcohol compared with CQ. Composites containing CQ and PPD showed similar results, except for depth of cure and hardness of bottom surface.

**Conclusion** BAPO and MAPO showed higher flexural strength, elastic modulus, hardness on top surface, and lower polymer plasticization to CQ.

## Introduction

Resin composites are the most widely used direct restorative materials currently due to their easy handling and esthetic characteristics. Bisphenol A-glycidyl methacrylate (BisGMA) is the monomeric system of most composites used, but due to its high molecular weight, it shows high viscosity, low wettability, reduced capacity for incorporating filler particles, and a lower degree of conversion (DC). 1 To minimize these deficiencies, it is necessary to incorporate diluent monomers into organic matrix, most often triethylene glycol dimethacrylate (TEGDMA) to achieve the ideal viscosity, increase the concentration of filler particles, and improve DC. 1 2 3 Other methacrylates such as urethane dimethacrylate (UDMA) and BisEMA (bisphenol A dimethacrylate ethoxylate) can also be used in combination with BisGMA in different proportions, with variation in the percentage and size of filler particles. 4

Photoinitiator systems can be classified based on mechanism of formation of free radicals; in type I, the radicals are generated by fragmentation of photoinitiator molecule after the absorption of light, and in type II, the photoinitiators are excited by light and need to interact with a co-initiator for the formation of free radicals. 5 Most widely used photoinitiator in composites is camphorquinone (CQ) combined with a co-initiator, usually a tertiary amine that does not absorb light. 3 6 7 Curing device emits light activating the CQ that interacts with co-initiator, producing free radicals the initiate the formation of polymer chains. 8 However, this photoinitiator system has some disadvantages such as the yellowish color that can interfere with the final esthetic, 9 10 showing the highest color change values. 11 Aternative photoinitiators such as phenyl-propanedione (PPD), mono(acyl)phosphine oxide (MAPO), and bis(acyl)phosphine oxide (BAPO) have been researched, which could improve the quality of composites mainly in relation to coloring and DC. 6 The advantage of these photoinitiators is to react directly with the action of light curing unit, unlike the CQ that needs of a co-initiator, inducing direct effects on mechanical and optical properties of resin-based material. 12 In addition, alternative photoinitiators are more reactive compared with CQ, producing more free radicals capable of initiating the polymerization reaction; meanwhile, they show lower transmittance, resulting in increased DC only on top surface of resin-based material. 13

Adequate polymerization is extremely important for clinical success of composite restorations. This procedure requires an appropriate wavelength to activate the photoinitiator and initiate the process of converting monomers into polymers. A low DC reduces the mechanical properties and color stability of dental composites. 6 These alternative photoinitiators can be used effectively in resin composites, but they absorb wavelength close to 400 nm, visible ultraviolet region (UV-vis), and shorter than QC that is in 470 nm range. 6 7 14 Thus, multiple-emission peak light-emitting diode (LED) devices that reach the two regions of light absorption spectrum, approximately 467 and 400 nm, are recommended for non-CQ initiators. 14

These alternative initiators with maximum absorption in UV region, which do not require a co-initiator, they could decrease the exposure time to light and increase the polymerization and mechanical properties with lower concentrations of photoinitiator. In addition, CQ reduction would decrease the polymerization inhibition by oxygen. 5

Therefore, the purpose of this study was to assess the effect of different photoinitiator systems on Knoop hardness (KH), plasticization (P), depth of cure (DoC), flexural strength (FS), and elastic modulus (EM) of experimental resin composites. The experimental hypothesis tested was that the alternative photoinitiators would not affect the mechanical properties of experimental composites.

## Materials and Methods

### Preparation of Experimental Composites

Experimental resin composites were formulated by using a blend of dimethacrylate monomers, 50 wt% BisGMA (Sigma-Aldrich Inc., St Louis, Missouri, United States) and 50 wt% TEGDMA (Sigma-Aldrich Inc.) 7 15 and silanized filler particles corresponded to 65 wt% composites, 16 where the proportion was 13 wt% silica of 0.04 µm (Schott AG, Mainz, Germany) and 52 wt% barium aluminum silicate (BaAlSi) of 0.7 µm (Esstech Inc., Essington, Pennsylvania, United States). 17 The photoinitiators corresponded to 0.2 wt% CQ (Sigma-Aldrich Inc.) and co-initiator 0.8 wt% DMAEMA (dimethylaminoethyl methacrylate; Sigma–Aldrich Inc.) 15 18 ; 0.2 wt% phenyl propanolone (PPD; Sigma-Aldrich Inc.) and 0.8 wt% DMAEMA; 0.1 wt% CQ + 0.1 wt% PPD and 0.8 wt% DMAEMA 17 ; 0.42 wt% monoacylphosphine oxide (MAPO; Sigma-Aldrich Inc.) 15 ; and 0.50 wt% BAPO (bisacylphosphine oxide; Sigma-Aldrich Inc.). 17 Inhibitory agent used was 0.01 wt% BHT (2,6-bis (1,1-dimethylethyl)-4methylphenol; Sigma-Aldrich Inc.). 17

The components were weighed by using a precision scale (Shimadzu AX 200, Shimadzu Corporation, Tokyo, Japan). Initially, monomers were mixed by using a centrifuge (SpeedMixer DAC 150.1 FVZ-K; FlackTek Inc., Herrliberg, Germany), then the photoinitiator and inhibitor agents were added until homogeneous, finishing the mixture in centrifuge for 90 seconds at 2,500 rpm. Silica and BaAlSi fillers were added to organic matrix individually, and they mixed for 90 seconds at 2,500 rpm. Resin composites remained stored in lightproof containers to prevent the exposure to ambient light.

### Specimen Preparation

Specimens were light cured for 20 seconds by using a multiple-emission peak curing unit (Bluephase G2; Ivoclar Vivadent, Schaan, Liechtenstein) at irradiance of 1,200 mW/cm monitored by radiometer. The LED used reaches two bands of light spectrum in the ultraviolet (UV) band (380–420 nm) and in the blue band (420–490 nm) reaching a total range of 380–490 nm. 17 Experimental composite was inserted in a single increment inside the mold, a polyester strip, and glass plate were placed on upper portion and slightly pressed afterward the plate was removed before light curing in contact with the polyester strip. BioEstat 5.0 software was used to determine the number of samples in this study with significance level of 5% and test power of 90%, with a suggested number of 10 samples per group.

### Knoop Hardness and Plasticization

KH ( *n* = 10) was measured by using cylindrical specimens (4 mm in diameter × 2 mm in thickness). After light curing, specimens were stored dry in a light-proof container for 24 hours, then specimens were submitted to indentation test (HMV-2T, Shimadzu, Tokyo, Japan). The long axis of Knoop indenter tip was positioned perpendicular to top and bottom surface of experimental composite. Three indentations were performed with a load of 50 g for 15 seconds. Specimens were stored in absolute ethanol for 24 hours, and KH was remeasured to obtain polymer P by hardness reduction after immersion in alcohol. 19

### Depth of Cure

For DoC by hardness profile, 10 specimens were made by using a semicircular mold of 4 mm in thickness and 2.5 mm of radius. Top surface was light cured, and the hardness was measured from the surface (contact with the light curing agent) at 0.1, 0.5, 1.0, 1.5, 2.0, 2.5, 3.0, 3.5, and 4.0 mm with 50 g load for 15 seconds. Hardness values of each depth were defined by the average obtained from three indentations; DoC was identified when the average hardness was 80% of initial hardness.

### Flexural Strength and Elastic Modulus

For 3-point bending testing ( *n* = 10) bar-shaped (7 mm in length, 1 mm in height, and 2 mm in width) specimens were prepared by using a silicone mold. After light curing, specimens were stored dry in a light-proof container for 24 hours, then specimens were placed in a device with two parallel supports separated by a distance of 5 mm of a universal testing machine (model 3382; Instron, Norwood, Massachusetts, United States) at cross-head speed of 1 mm/min, applied in center of specimen with continuous load up to fracture limit. FS was obtained in MPa (MegaPascal) by formula: FS = 3 *Fd* /2 *l* a ^2^ ; where *F* is the maximum load force (N), *d* is the distance between the supports (mm), *l* is the specimen width (mm), and a is the specimen thickness (mm). EM was obtained in GPa (GigaPascal) by formula: EM = ( *Fd* ^3^ /4 *la* ^3^ *D* )×10 ^−3^ ; where *F* is the maximum load (N), *d* is the distance between the supports (mm), *l* is the specimen width (mm), *a* is the specimen thickness (mm), and *D* the specimen deflection (mm).

### Statiscal Analysis

Kolmogorov–Smirnov and Levene tests were used to verify the data distribution in normality and homogeneity, respectively. One-way analysis of variance (ANOVA) followed by Tukey’s post hoc test were used at the significance level of 5%.

## Results

### Knoop Hardness and Plasticization

Top surface showed higher KH compared with bottom surface for all experimental composites ( *p* < 0.05). Composite containing MAPO showed the highest KH on top surface among all restorative materials followed by BAPO, which it was higher compared with CQ and PPD. These showed no significant difference between them ( *p* > 0.05), while CQ + PPD showed the lowest KH ( *p* < 0.05). For bottom surface, experimental composite containing BAPO showed the highest KH, which followed by CQ, CQ + PPD, MAPO, and PPD ( Table 1 ); all materials with significant difference among them ( *p* < 0.05).

**Table 1:** Means (standard deviation) of Knoop hardness (KgF/mm ^2^ ) of experimental composites according initiator system and surface analyzed

Initiator system	Surface	
	Top	Bottom
CQ	42.01 (0.60) ^c, A^	35.66 (1.01)b, B
PPD	41.42 (0.77) ^c, A^	25.65 (0.94) ^e, B^
CQ + PPD	40.59 (0.23) ^d, A^	32.40 (0.31) ^c, B^
MAPO	49.56 (0.24) ^a, A^	28.42 (0.53) ^d, B^
BAPO	46.55 (0.30) ^b, A^	37.37 (0.53) ^a, B^
Abbreviations: BAPO, bis(acyl)phosphine oxide; CQ, camphorquinone; MAPO, mono(acyl)phosphine oxide; PPD, phenyl-propanedione. Note: Distinct letters (lowercase comparing photoinitiator within each surface and uppercase comparing surface within each photoinitiator) indicate statistically significant difference ( *p* < 0.05).		

Hardness reduction after alcohol immersion on top surface was higher compared with bottom for all restorative materials ( *p* < 0.05), except to BAPO-based composite in which the *p* -values were statistically similar ( *p* > 0.05). Experimental composites containing CQ and PPD showed the highest polymer P on top surface, which followed by CQ + PPD, while MAPO and BAPO showed the lowest P ( *p* < 0.05). For bottom surface, P was higher for BAPO and CQ, these without significant difference between them; followed by CQ + PPD, MAPO, and PPD ( *p* < 0.05; Table 2 ).

**Table 2:** Means (standard deviation) of polymer plasticization (%) of experimental composites according initiator system and surface analyzed

Initiator system	Surface	
	Top	Bottom
CQ	33.37 (1.36)a, A	23.08 (1.63)a, B
PPD	34.21 (1.53)a, A	9.55 (1.88)d, B
CQ + PPD	31.25 (1.38)b, A	17.98 (0.61)b, B
MAPO	23.12 (1.22)c, A	11.81 (1.81)c, B
BAPO	22.78 (1.53)c, A	23.26 (1.06)a, A
Abbreviations: BAPO, bis(acyl)phosphine oxide; CQ, camphorquinone; MAPO, mono(acyl)phosphine oxide; PPD, phenyl-propanedione. Note: Distinct letters (lowercase comparing photoinitiator within each surface and uppercase comparing surface within each photoinitiator) indicate statistically significant difference ( *p* < 0.05).		

### Depth of Cure

Experimental composite using PPD as initiator showed DoC at 1.5 mm, in which the hardness was 32.92 kgF/mm ^2^ , by reduction of hardness profile in 80% (32.89 kgF/mm ^2^ ) of top surface (0.1 mm: 41.12 kgF/mm). Restorative materials containing BAPO and CQ+PPD also showed the highest DoC at 1.5 mm. Composite formulated using CQ showed the highest DoC at 2 mm. In composite containing MAPO, the DoC was 1.0 mm from top surface; it was the lowest among the experimental resin composites ( Table 3 ).

**Table 3:** Depth of cure (mm) of experimental composites according initiator system

Initiator system	Knoop hardness (KgF/mm ^2^ )						Depth of cure	80% KH reduction
	0.1 mm	0.5 mm	1.0 mm	1.5 mm	2.0 mm	2.5 mm		
CQ	42.33	40.12	38.82	37.66	35.18	31.75	2.0 mm	33.86
PPD	41.12	37.72	35.77	32.92	28.76		1.5 mm	32.89
CQ + PPD	40.62	38.31	35.96	34.61	32.33		1.5 mm	32.49
MAPO	49.18	46.59	43.63	39.06			1.0 mm	39.35
BAPO	46.65	44.32	43.16	40.08	36.15		1.5 mm	37.32
Abbreviations: BAPO, bis(acyl)phosphine oxide; CQ, camphorquinone; KH, Knoop hardness; MAPO, mono(acyl)phosphine oxide; PPD, phenyl-propanedione.								

### Flexural Strength and Elastic Modulus

FS and EM values of experimental composites are shown in Table 4 . Composite containing MAPO showed higher FS values compared with experimental materials containing CQ and PPD ( *p* < 0.05), these showed no significant difference between them ( *p* > 0.05). BAPO and CQ + PPD-based restorative materials showed intermediate means, without significant difference between them and with others experimental composites ( *p* > 0.05).

**Table 4:** Means (standard deviation) of flexural strength (MPa) and elastic modulus (GPa) of experimental composites according initiator system

Initiator system	Flexural strength	Elastic modulus
CQ	126.43 (14.75)b	3.33 (0.18)c
PPD	123.39 (9.32)b	3.28 (0.12)c
CQ + PPD	132.08 (15.39)ab	3.75 (0.15)b
MAPO	148.56 (12.19)a	4.36 (0.18)a
BAPO	132.08 (13.57)ab	3.77 (0.29)b
Abbreviations: BAPO, bis(acyl)phosphine oxide; CQ, camphorquinone; MAPO, mono(acyl)phosphine oxide; PPD, phenyl-propanedione. Note: Different letters indicate a statistically significant difference within each mechanical test, comparing the experimental groups ( *p* < 0.05).		

Composite resin containing MAPO as photoinitiator system showed the highest EM ( *p* < 0.05), followed by composite containing BAPO and CQ + PPD, these showed no significant difference between them ( *p* > 0.05). While CQ- and PPD-based restorative materials showed the lowest EM values ( *p* < 0.05), these showed no significant difference between them ( *p* > 0.05).

## Discussion

The photoinitiator of greatest use since light cured resin composites were developed is CQ-amine. 7 17 20 Laboratory tests widely used for FS and EM follow the International Organization for Standardization (ISO) 4049 standard; however, changes in these parameters are commonly reported in literature, mainly in relation to the specimen size. 10 21 Specimen with smaller size reduces costs and facilitates its preparation. In addition, the specimen is light cured in a single moment, which justifies its greater employability in most recent studies. 22

FS is considered a clinically important property to assess restorative materials that will be used in areas of high occlusal strength. 23 A 3-point bending is the test of easy application that could be correlated clinically to simulate failures caused by high tensions, arising from occlusal forces. 24 The limit established in ISO 4049 standard for FS test is 80 MPa for polymer-based restorative materials used in occlusal surfaces. 25 In the present study, all experimental composites showed higher FS than this limit. Although it is not possible to correlate a laboratory test with clinical use, some authors consider 130 Mpa as an ideal value for flexural strength of composite resins. 26 In the present study, CQ and PPD were the only groups that presented lower values, the other groups of CQ + PPD, MAPO, and BAPO presented higher values for FS.

Corroborating with the finding of this investigation, a previous study 15 observed higher FS and EM for a composite containing MAPO compared with CQ due to higher conversion of monomer to polymer, improving the polymer quality. However, another study 27 observed similar FS values when the experimental restorative materials were formulated by using CQ and MAPO as photoinitiatior agents. Alternative initiators, including BAPO and CQ, also showed similar FS values in a previous investigation. 28 In these studies, a higher EM for MAPO 15 27 and BAPO 28 was observed. BAPO has been related to produce a higher number of free radicals by high reactivity, causing its DC to be higher compared with CQ. 28

The FS and EM were similar comparing experimental composites formulated with CQ-amine, PPD, and CQ + PPD by mini-flexural test. 29 Regarding FS, the results were the same as those found in this investigation for CQ, PPD, and CQ + PPD. However, the EM was higher for the combination of CQ + PPD than CQ and PPD photoinitiators used alone. This fact attributes to CQ and PPD having similar chemical structure, provide mechanical characteristics, and DC. 29

It was reported that experimental composites formulated using CQ, PPD, and CQ/PPD photoinitiators resulted in similar KH values on top surface, regardless of light curing protocol. 29 However, different results were observed in the present study, in which CQ and PPD were similar and CQ/PPD combination showed the lowest KH values compared with other composites on top surface. On bottom surface, CQ showed higher KH compared with CQ/PPD, which it was higher than PPD initiator. These results could be explained by higher DoC observed for CQ (2 mm) and also because the PPD requires UV light for its activation and this cannot penetrate very deeply into the material. 13

MAPO based experimental composite had the highest hardness values on top surface compared with CQ. 27 Composite containing BAPO was reported to have higher KH for top and bottom surfaces, while MAPO and CQ showed similar KH values for both surfaces. 30 In another study, MAPO and BAPO showed similar KH values on top and bottom surfaces, but these values were higher compared with CQ on both surfaces. 20 In this investigation, MAPO showed higher KH than BAPO, followed by CQ on top surface. Inversely, on bottom surface MAPO showed the lowest KH values, followed by CQ and BAPO. Differences in photoinitiator concentration and light curing protocol can explain the conflicting results. Although initiator alternatives have a higher reactivity, the lower transmittance impairs deep polymerization. 13

In this investigation, the KH values were higher on top surface for all groups, corroborating with previous studies. 20 30 Hardness reduction of bottom surface is related with a decrease in number of photons reaching the deepest region of composite. When the light passes through the material structure, the irradiance is reduced, while on top surface the light arrives with least attenuation, allowing higher energy for excitation of photoinitiator molecules. 31 32 Hardness change of composites is also associated with translucency of resin matrix, which depend of thickness, size and type of filler particles, pigments, and light absorption capacity; factors that affect dispersion of light inside the composite for both violet and blue light transmission. 13 33 The intensity and quality of the photopolymerization unit interfere with color stability and microhardness, influencing the longevity of the restoration. 34

Hardness reduction after alcohol immersion has been used to assess the polymer structure of resin material, indicating the polymer plasticization and used as indirect method to estimate the crosslink density. 19 35 A significant KH reduction at both top and bottom surfaces for all experimental composite was observed. The degree of decomposition is affected by characteristic of resin matrix of each material and by temperature and type of substance used as a solvent. 35 The resin composition has an impact on the solubility behavior of resin composites. 36 The greater the bonding between the components of polymer, the greater its resistance to plasticization by solvent action. That is because the closer the particles are inside the matrix, the lower solution would be able to penetrate its structure and change its characteristics. 19 The organic matrix, the filler particle and the polishing of the restorations can increase the physical properties making it more resistant to degradation. 37

Inadequate polymerization of adhesive resin-based restorations has been related to clinical failures due to a greater chance of fracture, secondary caries, and/or premature wear. 35 38 39 Overall, DoC is evaluated according to ISO 4049 standard, but comparing the ISO standard with hardness profile, it was found that there is an overestimation in relation to the DoC. 40 41 In this way, the use of Knoop or Vickers hardness profile would be recommended for adequate DoC characterization of resin composites, avoiding future clinical problems. 35

In a previous investigation, 20 following the ISO 4049 standard, it was found that experimental composites containing BAPO, MAPO, and CQ as initiator agent showed DoC of 3.6, 3.2, and 3.7 mm, respectively. In our study, DoC was found above these values, which were 1.0 mm for MAPO, 1.5 mm for BAPO, and 2.0 mm for CQ. These values can be justified by difference in methodology and possible overestimation of ISO 4049 standard.

Alternative photoinitiators commonly absorb violet light, which has a lower transmittance than blue light. 13 So, despite MAPO and BAPO exhibit higher DC compared with CQ, 13 18 in another study MAPO showed lower DC. 28 An important characteristic for resin composites is their polymerization efficiency, since the increased DC leads to an improvement in some physical and mechanical properties of restorative material. 6 Thus, the use of incremental filling of composites containing more efficient photoinitiator systems combined to light curing at high irradiance, impacts in enhanced resultant polymer structure. 42

New photoinitiator systems capable of optimizing the limitations of current photoinitiators in terms of curing efficiency, aesthetics, physicochemical properties, and biocompatibility are very important for the development of materials. MAPO and BAPO, in addition to these advantages, allow their use in conjunction with pigmented photoinitiators in smaller quantities, maintaining or improving the qualities of the material. 7 Therefore, since the alternative photoinitiators affected the mechanical properties of experimental composites, the experimental hypothesis was accepted.

The limitations of this laboratory study do not equate to clinical studies. Another limitation would be the inability to control the characteristics of the environment such as humidity and temperature at the time of the tests. Further studies with variations in the proportions of photoinitiators, resin matrix, and filler particles would be interesting to find the composition with the best physical chemical property of the material.

## Conclusion

It can be concluded that the top surface showed higher hardness compared with bottom for all composites; CQ showed the highest depth of cure by hardness profile. Overall, BAPO and MAPO showed higher flexural strength, elastic modulus, hardness on top surface, and lower polymer plasticization caused by alcohol on top surface.
